# Health Tract No. 349

**Published:** 1868-11

**Authors:** 


### HEALTH TRACT NO. 349.

### Healthful Atmosphere.

There are many ingredients in the air we breathe which render it impure, but the most controlling one perhaps is carbonic acid gas. The purest atmosphere has about four parts in ten thousand, and to that extent, is no doubt, a healthful ingredient; but in proportion as it is increased it becomes unhealthful, dangerous, and deadly. The burning of charcoal produces carbonic acid gas so rapidly, that if a person goes to sleep in a close room where a few lumps of it are burning in an open vessel, he will be found dead in the morning. If a gaslight is burning in a close room, thirteen feet square and five persons are sleeping there, the amount of carbonic acid gas is doubled in seven minutes, this proves that the act of breathing produces carbonic acid gas, that a person generates carbonic acid gas all the time, the amount being increased in proportion to the number of persons in the apartment, as in churches, theatres and the like. Physicians have closely investigated these matters and the results they give are very impressive. The mid-Atlantic and the high mountains give four, the great cities of the world six parts of carbonic acid in ten thousand parts of air; but if we come into houses, it is a very different thing. A stable has seven parts to begin with; no wonder a consumptive person got well by living in a cow-house; no wonder the scavengers of Paris, the dirtiest wretches in the world, have better health than its titled ladies, the scavengers live in the open air! A ball-room has fifty-six parts. At the beginning of an hour's lecture M. Dumas' room had forty-two parts, at the close sixty-seven parts in ten thousand; a well filled school-room is the worst of all, having seventy-two; the air of a bed-room at rising in the morning has forty-eight; after two hours' ventilation sixteen, showing strongly the importance of opening the doors and hoisting the windows of our chambers, after we leave them. There is no use then in going to the country, the springs or the sea shore in the summer, if we hang around the house and lounge on its sofas or loll in its chairs, the benefit to be derived is in proportion as we breathe the out door air in the employment of interested activities. Undulating, elevated situations are more healthful than level low lands because drier. The most healthful atmosphere in a sitting room is obtained by having a blazing fire on the hearth and an open door; and the same at night for a chamber is far more healthful than to sleep in a cold close room. A small warm room with even a small air hole is more healthful than a very large close cold room; cold air is not necessarily a pure air and no person can be well long, who is not in the open air, more or less every day.

